# Weight management interventions for adults living with overweight or obesity and severe mental illness: a systematic review and meta-analysis

**DOI:** 10.1017/S0007114522003403

**Published:** 2023-08-14

**Authors:** Heidi Stevens, Jo Smith, Lauren Bussey, Alison Innerd, Grant McGeechan, Sarah Fishburn, Emma Giles

**Affiliations:** 1 School of Health and Life Sciences, Teesside University, Southfield Road, Middlesbrough TS1 3BX, UK; 2 Tees, Esk and Wear Valleys NHS Foundation Trust, Flatts Lane Centre, Flatts Lane, Normanby, Middlesbrough TS6 0SZ, UK; 3 School of Social Sciences, Humanities and Law, Teesside University, Southfield Road, Middlesbrough TS1 3BX, UK

**Keywords:** Overweight, Obesity, Severe mental illness, Weight management, Effectiveness, Systematic review

## Abstract

When compared with the general population, people living with severe mental illness (SMI) are 1·8 times more likely to have obesity while in adult mental health secure units, rates of obesity are 20 % higher than the general population. In England, there are currently 490 000 people living with SMI. The aim of this systematic review was to collate and synthesise the available quantitative and qualitative evidence on a broad range of weight management interventions for adults living with SMI and overweight or obesity. Primary outcomes were reductions in BMI and body weight. Following sifting, eighteen papers were included in the final review, which detailed the results of nineteen different interventions; however, there was a lack of qualitative evidence. Pooled results for three studies (MD − 3·49, 95 % CI − 6·85, −0·13, *P* = 0·04) indicated a small effect in terms of body weight reduction but no effect on BMI for four studies (MD − 0·42, 95 % CI − 1·27, 0·44, *P* = 0·34). Key recommendations for future research included integration of qualitative methodology into experimental study design, a review of outcome measures and for study authors to follow standardised guidelines for reporting to facilitate complete and transparent reporting.

Overweight and obesity are defined as excessive fat accumulation that may impair health^(1)^. The WHO^(1)^ defines overweight as a BMI greater than or equal to 25 kg/m^2^ and obesity as a BMI greater than or equal to 30 kg/m². Overweight and obesity have increased in the UK since 1993^(2)^; currently, an estimated 66·9 % of men and 59·7 % of women in England are living with overweight or obesity^(3)^. Similarly, 60 % of adults in Wales and 66 % of adults in Scotland have overweight or obesity^(4,5)^. Overweight and obesity are non-communicable diseases that may increase the risk of premature mortality or co-morbidities such as CVD, type 2 diabetes and certain cancers. People with severe mental illness (SMI) are 1·8 times more likely to have obesity than the general population^(6)^, and obesity rates in mental health secure units are 20 % higher than in the general population^(7)^.

SMI includes the most serious mental health conditions that share basic characteristics including significant symptom severity, severe functional impairment and an enduring impact on a person’s daily life, defined as conditions related to schizophrenia, psychosis and bipolar disorder^(8–10)^. In England alone, there are 490 000 people living with SMI^(11,12)^.

The underlying reasons for overweight and obesity in SMI are not fully understood but include complex preventable risk factors such as poor diet, reduced physical activity and emotional eating, which often stems from feelings of worthlessness^(13–15)^. Access to healthy food is an issue in both inpatient and community settings^(15)^. Inpatients are reliant on hospital food which can often be unhealthy and inappropriately portioned^(15)^, while affordability can be a barrier to healthy food for community patients^(16,17)^. Evidence has shown that limited physical activity opportunities for inpatients may result in increased sedentary behaviour^(15,18)^, and in the community people with SMI are more sedentary than the general population^(19,20)^. Sedentary behaviours such as sitting and lying down^(21)^ have been linked to increased odds of obesity particularly in terms of screen-based entertainment^(22)^. Often antipsychotics can also have a sedative effect on patients, while some atypical antipsychotics, primarily olanzapine and clozapine, can also cause a lack of satiety which increases the risk of weight gain^(14,20)^.

National strategies have aimed to improve mental health services in England, but challenges in system-wide implementation and the increasing prevalence of mental illness have resulted in inadequate services and worsening outcomes^(11)^. In 2014/15, two million adults made contact with specialist mental health services and 90 % of adults with SMI accessed community services^(11)^; however, there are variations in the implementation of existing weight management guidance for people with SMI^(7)^. National Institute for Health and Care Excellence (NICE) guidance inpatient management includes regular assessment of BMI, medication and lifestyle behaviours^(7)^, while more recent guidance endorses holistic and personalised approaches led by service users, for example, coproduced physical heath passports^(23)^. Physical heath passports enable service users to set and monitor their own nutrition, physical activity and psychological needs^(23)^. Further general guidelines for the commissioning of community Tier 2 Adult Weight Management Services recommend that services be multi-component while adhering to government dietary guidelines^(24)^ and for those with mental illness, psychological therapies are also recommended when adapting weight management services^(25)^.

The National Institute for Health and Care Research (NIHR)^(26)^ recommends Patient and Public Involvement (PPI) as a rich source of information for making health research more patient-centred. However, recent systematic reviews on weight management and SMI are restricted to randomised controlled trials (RCT)^(27–34)^. Furthermore, they focus on a single element of weight management or a single diagnosis^(27,32,35,36)^. Some reviews include obesity^(33)^ but not overweight, include participants with a healthy weight^(27,37)^, or do not stipulate weight or BMI thresholds^(29,30,32,38)^. This systematic review takes a broader approach, including a range of diagnoses, interventions and settings. A mixed methods approach was used in an attempt to capture both experimental data and the lived experience of participants.

## Aim

The aim of this systematic review was to collate and synthesise the available quantitative and qualitative evidence on a broad range of weight management interventions for adults with SMI and overweight or obesity.

### Review questions:

Are weight management interventions effective for adults with overweight or obesity and SMI?

Which elements of weight management interventions are effective with overweight or obesity and SMI?

What is the acceptability of weight management interventions for adults with overweight or obesity and SMI?

## Methods

### Data sources and search strategy

This systematic review followed the PRISMA twenty-seven-item checklist for transparent reporting of systematic reviews of healthcare interventions (Appendix 1)^(39)^. The search strategy and protocol were published in the PROSPERO International Prospective Register of Systematic Reviews (registration number: CRD42021235318). Guidance was sought from an information scientist to generate the search strategy. The following databases were searched in February 2021 and in May 2022 for qualitative, quantitative or mixed methods primary studies evaluating interventions for weight loss in adults with overweight or obesity and SMI: AMED, CINAHL, Medline complete, Embase and Web of Science. The search strategy included a combination of key words and terms related to ‘severe mental illness’ and ‘overweight’ or ‘obesity’ (Appendix 2).

### Eligibility criteria

We included papers published in English reporting on participants 18 years and above, with overweight or obesity (BMI greater than or equal to 25 kg/m^2^) and a diagnosis of SMI. SMI included conditions related to schizophrenia, psychosis and bipolar disorder. We included weight management interventions involving elements of physical activity, pharmacology, diet, food and nutrition, healthy lifestyle, psychology, education, information giving and/or support. Following discussions with mental health clinicians, it was concluded that surgical interventions were out of the scope of this review as such services are provided by tier 4 weight management services in the acute sector. Comparator groups (CG) were treatment as usual (TAU), no care or an alternative intervention (any other weight management intervention).

To assess effectiveness, the primary outcomes of interest were change in BMI and body weight with secondary outcomes including changes in waist circumference, or/and body composition, quality of life (QoL), perceived impact on mental health and attrition. Studies were included if they were RCT or quasi-RCT. To assess acceptability, qualitative studies included focus groups, interviews or surveys. Non-English language papers were excluded as we lacked the capacity for translation.

### Selection process

In stage one sifting, a reviewer (HS) screened all titles, subject headings and abstracts for key words guided by population, intervention, and study design. Full-text articles were obtained for eligibility assessment, and the reviewer screened all full-text articles for inclusion using Rayyan QCRI. Six independent reviewers (ELG, GJM, LB, JS, AI and SF) double-sifted all papers at each stage, and disagreements were resolved through discussion. Results and consensus were recorded on a Microsoft Excel spreadsheet.

### Data collection process

Data from the included studies were recorded on a pre-piloted data extraction sheet. One reviewer extracted data for all included papers (HS) and four independent reviewers (ELG, GJM, LB and AI) checked extracted data, with disagreements resolved through discussion. Items extracted included SMI diagnosis, age, sex, setting, intervention components, outcomes measures and drop-out rates.

### Quality appraisal

Quality appraisal of the conduct and reporting of included studies was assessed using Critical Appraisal Skills Programme (CASP) checklists for study design^(40)^. One reviewer (HS) assessed all papers and four independent second reviewers (AI, ELG, GJM and LB) checked the appraisals independently, with disagreements resolved through discussion.

### Data synthesis

Mean differences (MD) and 95 % CI were calculated to compare differences in treatment effects between intervention and comparison for BMI (kg/m^2^) and weight (kg). Review Manager software (RevMan 5.3) was used to conduct meta-analyses where appropriate and results graphically displayed as forest plots^(41)^. Effect size was judged as 0·8 a large effect, 0·5 a moderate effect and 0·2 a small effect. Statistical heterogeneity was assessed using the *χ* ^2^ test (*P* = 0·1) and quantified using I^2^ statistic as per Cochrane Collaboration Guidelines^(42)^. Sensitivity analysis was to be conducted by removing any study that had potential issues with bias^(42)^. Where possible, subgroup analyses were to be performed to explore potential sources for heterogeneity^(42)^ by category of intervention, setting or SMI diagnosis.

An inductive, thematic analysis approach was pre-specified for extracted qualitative data to identify codes and develop themes that potentially address the review questions^(43)^. The final master themes and quantitative results were to be synthesised in a mixed method approach.

## Results

### Study selection

**Fig. 1.**
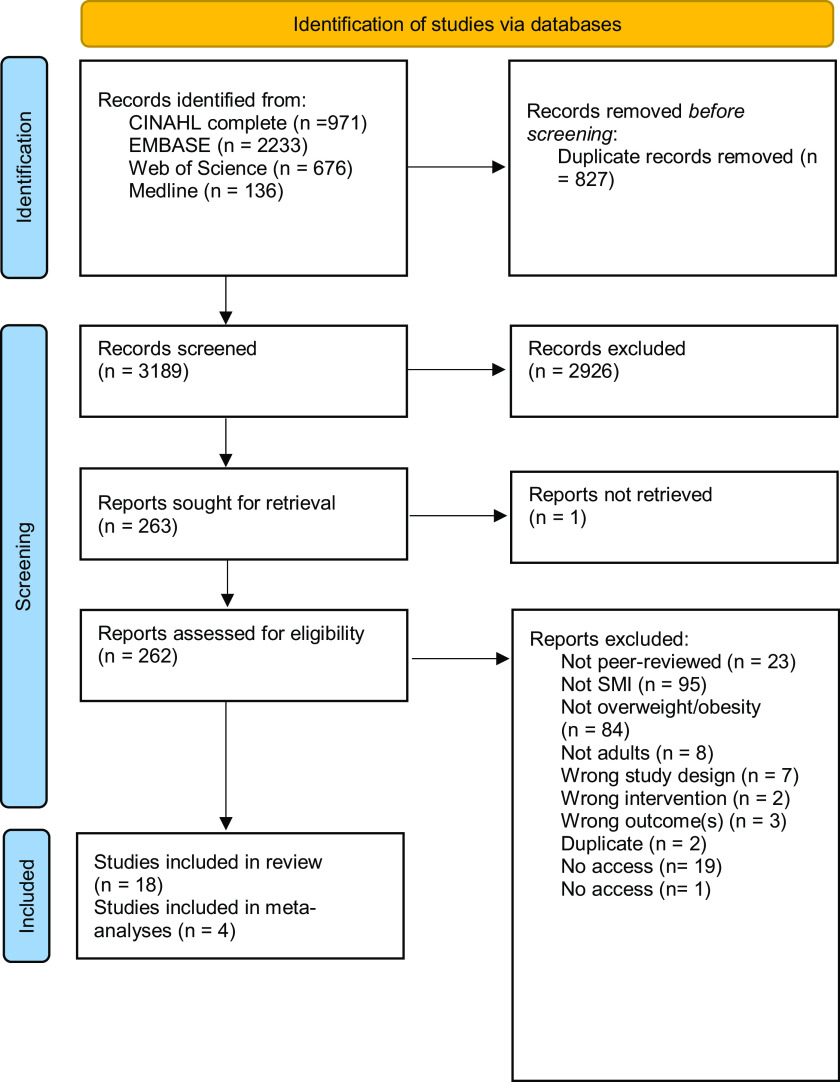
PRISMA flow chart.

Identification of studies and reasons for exclusion are illustrated in Fig. 1. Following searches of scientific databases in February 2021, 3408 studies were identified, after removal of duplicates, 2581 records were screened of which 233 full-text articles were reviewed to determine eligibility and sixteen included in the review. In May 2022, a further rerun identified 608 studies of which twenty-nine full-text articles were screened for eligibility and two included in the review. A record of excluded studies can be found as supplementary material with this article.

### Study characteristics

Following sifting, eighteen papers were included in the final review, which detailed the results of nineteen different interventions^(44–59)^ (see Table 1 for study characteristics). Two studies included two intervention groups in addition to a CG^(49,56),^ and Henderson *et al.* ^(51)^, compared two pharmacology interventions with no CG. Included studies were from Canada (*n* 2)^(44,53)^, Italy (*n* 2)^(45,49)^, Japan (*n* 1)^(56)^, New Zealand (*n* 1)^(47)^, Spain (*n* 1)^(55)^, Taiwan ROC (*n* 2)^(54,59)^, Turkey (*n* 1)^(57)^, the UK^(60,61)^ and the USA (*n* 6)^(46,48,50–52,58)^. Studies were RCT (*n* 10)^(45,47,48,50,55–59,61)^ and quasi-experimental studies (*n* 7)^(44,46,49,51–54)^, and one was a nested qualitative study^(60)^ of an included RCT^(61)^. The total number of participants were *n* 1312 ranging from 17^(58)^ to 332^(55)^ study participants. Baseline BMI values ranged from 28·55 kg/m^2^ which is classed the overweight category to 44·9 kg/m^2^ which is classed as the obese type 2 category^(1)^. The weight of participants ranged from 75·5 kg to 117 kg.

**Table 1.** Table of characteristics and within-study results for all studies included in the systematic review

Author (year, country)	Study design	Baseline participants (n)	Mean age (years)	Sex	SMI diagnosis	Intervention	Comparison	Outcome measure(s)	Within-study results
Battaglia *et al.* (2013) (Italy)^(45)^	RCT	*n* 23 IG *n* 12 CG *n* 11	IG 36·00, sd 5·00 CG 35·00, sd 4·00	100 % male	Schizophrenia and/or schizoaffective disorder	Soccer training: training twice weekly. Technical–tactical strategies to promote a combination of moderate- and vigorous-intensity activity. Setting: unreported	Instructed not to engage in physical activity.	BMI (kg/m^2^) Wt QoL	Follow-up 12 weeks. IG = 10; CG = 8 IG – significant within-group decrease in BMI and wt (*P* = 0·001). CG – significant within-group increase in BMI and weight (*P* = 0·05). Between groups change in SF-12 PCS and SF-12 MCS in favour of TG (*P* < 0·001). Adherence: > 80 %
Elmslie *et al.* (2006) (New Zealand)^(47)^	RCT	*n* 60	IG 42, sd 10 CG 42, sd 13	*n* 49 (81·7 %) female	Bipolar disorder	Carnitine supplementation: individualised diet plan, physical activity 5 days per week. Lifestyle advice provided by a dietitian. Setting: community	Placebo capsules. Diet plan, physical activity and lifestyle advice same as intervention group.	BMI (kg/m^2^) Wt Waist circumference Mood – Montgomery–Asberg Depression Rating Scale (MADRS).	Follow-up 26 weeks. Participants *n* 44 No significant difference between groups for change in BMI (*P* = 0·466), wt (*P* = 0·381) or waist circumference (*P* = 0·597). No significant difference between groups for MADRS (*P* = 0·190). Adherence: unreported.
Frank *et al.* (2015) (USA)^(48)^	RCT	*n* 122 IG *n* 61. CG *n* 61.	41·6, sd 9·5 IG 41·8, sd 9·5 CG 41·4, sd 9·7	Male and female	Bipolar disorder	Psychopharmacologic management. The Healthy Lifestyle Program: 15 individual sessions. Psychoeducation, wt loss nutrition, physical activity. Setting: outpatients	Psychopharmacologic management without Healthy Lifestyles Program.	BMI (kg/m^2^)	Follow-up 14 weeks, Participants *n* 109 IG mean BMI decrease of 2·3 % (sd 3·8). CG mean BMI decrease of 0·2 % (sd 3·9), significant difference between groups in favour of IG (t = 2·8, *P* = 0·006). Adherence: unreported.
Henderson *et al.* (2005) (USA)^(50)^	RCT	*n* 37 IG *n* 19 CG *n* 18	IG 3·2, sd 10·6 CG 40·7, sd 9·9	IG: 7 (37 %) female CG: 7 (39 %) female	Schizoaffective disorder, schizophrenia	Sibutramine: weekly group/individual sessions focused on nutrition, exercise, behavioural modification and goal setting. Setting: outpatient clinic of an urban mental health centre	Placebo capsules Individual and group sessions identical to intervention group.	BMI ((kg/m^2^) Wt Waist circumference Body composition Global Assessment Scale, Positive and Negative Syndrome, Scale for the Assessment of Negative Symptoms.	Follow-up 12 weeks Participants *n* 31 Significant between groups difference in BMI (*P* < 0·05), wt (*P* < 0·05) and waist circumference (*P* < 0·005) in favour of IG. Significant increase in waist:hip ratio in IG (*P* < 0·05). Adherence: unreported.
Masa-Font *et al.* (2015) (Spain)^(55)^	RCT	*n* 332 IG *n* 169 CG = 163	46·7	45 % female	Schizophrenia, schizoaffective, bipolar disorder	Educational and physical activity programme: education component. Physical activity, increase daily steps in local streets. Healthy dietary habits, diet diary. Setting: Mental Health Centres, local streets.	TAU, regular psychiatrist check-ups.	BMI (kg/m^2^) Waist circumference QoL SF-36 Mental health – Clinical Global Impression (CGI)	Follow-up 3 months IG *n* 169; CG *n* 163 Significant decrease in BMI in favour of the CG (*P* = 0·038). No significant differences in waist circumference. Adherence: 42·6 %
Sugawara *et al.* (2018) (Japan)^(56)^	RCT	*n* 265 Group A, *n* 85 Group B, *n* 93 Group C, *n* 87	Group A, 44·0 sd, 10·3 Group B, 47·6 sd, 9·6 Group C, 46·6 sd, 10·9	Group A, 57 % (35) male Group B, 46 % (31) male Group C, 53 % (32) male	Schizophrenia or schizoaffective disorder	Group B psychiatrist wt loss advice: body wt measurements taken at individual sessions with brief advice. Notebook to record progress. Group C nutrition education: same as Group B plus structured nutrition education sessions with dietitians once a month. Daily food records. Setting: outpatient	Group A, TAU for schizophrenia.	BMI (kg/m2) Wt Waist circumference	Follow-up 12 months Participants *n* 189 Within Group C, there was a significant decrease in wt (p = < 0·00), BMI (p = < 0·001) and waist circumference (*P* = 0·007 from baseline to follow-up but not in Group A or B. Adherence: unreported.
Urhan, Aksoy and Ayer (2015) (Turkey)^(57)^	RCT	*n* 30 IG *n* 15 CG *n* 15	IG 38·08 sd 4·79 CG 37·71 sd 6·04	100 % female	Schizophrenia	Wt loss diet therapy: nutrition and physical activity education. Personalised diet recipes. Dietary motivation. Exercise, daily 30 min walking at moderate pace. Food and physical activity diary. **Setting** inpatient	Same as IG (no diagnosis of Schizophrenia.)	BMI (kg/m2) Wt Waist circumference Body fat (%) Hip circumference Waist:hip ratio	Follow-up 8 weeks IG *n* 13; CG *n* 14 There were significant decreases in all anthropometric measurements within both groups (*P* < 0·05). Between groups CG had greater decreases in BMI, wt, waist circumference, body fat, hip circumference and waist:hip ratio Adherence: unreported.
Weber and Wyne (2006) (USA)^(58)^	RCT	*n* 17 IG *n* 8 CG *n* 9	Not reported	IG *n* 5 (62·5 %) female CG *n* 7 (77·8 %) female	Schizophrenia or schizoaffective disorder	Cognitive/behavioural group intervention: 60 min per week. Role playing, goal setting, problem-solving and discussions on barriers to change. Activities such as walking. Presentations on low-fat diets. Food and activity diaries. Setting: public mental health clinics.	TAU at the clinic. Weighed and measured (4-week intervals).	BMI (kg/m^2^) Wt Waist:hip ratio.	Follow-up 16 weeks IG *n* 9; CG *n* 7 Between groups the IG had greater wt reductions. Within groups no significant differences in wt, waist:hip ratio, or BMI scores. Adherence: unreported.
Whicher (2021) (UK)	RCT	*n* 47 IG *n* 24 CG *n* 23.	IG 43·9 sd 11·0 CG 45·4 sd 10·7	*n* 23 female	First episode psychosis, schizophrenia and schizoaffective disorder	Liraglutide as an adjuvant to promote wt loss. Setting: Mental health centres and primary care	Placebo	BMI Wt Waist circumference Impact on mental health	Follow-up 6 months IG *n* 15, CG *n* 19 Mean change Significant difference in favour of IG for BMI, weight and waist circumference.
Wu *et al.* (2005) (Taiwan (ROC)^(59)^	RCT	*n* 56 IG *n* 28 CG *n* 28.	IG 42·2 sd 7·5 CG 39·0 sd 6·7	IG *n* 17 female (61 %) CG *n* 14 (56 %) female	Schizophrenia	Diet and Physical Activity Programme: dietary assessment, including fruit and vegetables, sugar-free foods and drinks. Physical activity 3 d per week, walking up and downstairs. Rewards for participation Setting: Inpatients of a veteran’s hospital	No description reported	BMI Wt Body fat (%) Waist circumference Hip circumference Waist:hip ratio	Follow-up 6 months IG *n* 28, CG *n* 25 Significant difference in favour of IG for BMI, wt, waist circumference (*P* < 0·001) and hip circumference (*P* < 0·05). Adherence: unreported.
Abdel-Baki *et al.* (2013) (Canada)^(44)^	Quasi-experimental	*n* 25	25·9 (sd 3·9)	100 % male	Schizophrenia, schizoaffective disorder, bipolar disorder, other psychotic disorder	Aerobic interval training (AIT) twice weekly. High-intensity exercise training of shorter duration involving running and active recovery walks. Setting: unclear	N/A	BMI (kg/m^2^) Wt Waist circumference Lean mass (%) Muscle mass (%)	Follow-up 14 weeks Participants *n* 16 Significant decrease in waist circumference only (*P* = 0·015).
Centorrino *et al.* (2006) (USA)^(46)^	Quasi-experimental	*n* 22.	40·5 (sd 8·5) (*n* 17)	*n* 10 (58 %) female.	Schizophrenia or schizoaffective disorder	TRIAD: twice weekly meetings. Dietary counselling and exercise low-calorie food plan. One session every 4 weeks. Setting: weight management centre on hospital grounds	N/A	BMI (kg/m^2^) Wt QoL – SF-36/Quality-of-Life Questionnaire (QLS)	Follow-up 24 weeks, participants *n* 17 Significant decrease in BMI (*P* = 0·0005) and wt (*P* = 0008). SF-36 results not reported. QLS no significant differences at follow-up. Adherence: *n* 6 attended weekly sessions.
Galle *et al.* (2017) (Italy)^(49)^	Quasi-experimental	*n* 153 Interpersonal therapy *n* 50. Dialectical Behavioural Group *n* 50. TAU = 53	IT: 33, sd 4·21 (22–56) DBG: 34, sd 3·78 (26–47) CG: 32, sd 5·10 (21–48)	100 % female	Borderline personality disorder	12 months IT: evaluation of interpersonal relationships, 60-min weekly sessions, optional telephone consultation. DB: strategies for behaviour Modifications, optional telephone consultation. Setting: unreported	TAU Medical and psychological evaluation, meeting with bariatric surgeon.	BMI (kg/m^2^) Wt	Follow-up 12 months, participants *n* 139 Significant difference in BMI between groups in favour of IG group (*P* < 0·01). Wt outcomes only reported for subgroups. Adherence: > 65 %
Henderson *et al.* (2009) (USA)^(51)^	Quasi-experimental	*n* 24	Clozapine + ziprasidone 48, sd 7 Olanzapine + ziprasidone 52, sd 8	*n* 17 (81 %) male	Schizophrenia or schizoaffective disorder.	Ziprasidone: as an adjuvant to promote wt loss. Ziprasidone plus clozapine Ziprasidone plus olanzapine Setting: outpatient clinic, urban community mental health centre	No control	BMI (kg/m^2^) Wt Waist circumference Hip circumference QoL Positive and negative syndrome scale, Hamilton Depression Rating scale, Scale for the assessment of negative symptoms	Follow-up 6 weeks, participants *n* 21 No significant differences at follow-up for any outcomes. Adherence: unreported.
Katekaru, Minn and Pobutsky (2015) (USA)^(52)^	Quasi-experimental	*n* 47	31–60 (*n* 35) 61 and older (*n* 12)	15 (32 %) female	Paranoid schizophrenia, undifferentiated schizophrenia, schizoaffective disorder	Health counselling: repetitive behavioural counselling, motivational interviewing, physical activity and nutrition guidance. Weekly wellness classes. Setting: Community Mental Health Centre	N/A	BMI – (kg/m^2^)	Follow-up 5 years Participants *n* 47 Baseline: *n* 22 participants were obese (47 %), *n* 20 were overweight (43 %) Year 5: *n* 21 participants were obese (45 %), *n* 18 were overweight (38 %) Adherence: unreported.
Klam, McLay and Grabke (2006) (Canada)^(53)^	Quasi-experimental	*n* 19	26 to 62, median age = 41.	*n* 9 female	Schizophrenia or schizoaffective disorder	Personal Empowerment Program: a healthy lifestyle and wellness group, weekly sessions for 2 h. Education on nutrition, stress management and personal development. Exercise, core work and stretching. Community/affordable recreational activities. Food diary Setting: outpatient clinic	N/A	BMI (kg/m^2^)	Follow-up 38 weeks Participants *n* 16 Total wt loss was 149·6 pounds. Average BMI 37·55 kg at baseline and 36·84 kg/m^2^ at follow-up. Decrease in abdominal girth 20¾ inches. Adherence: average 80 %
Kuo *et al.* (2013) (Taiwan -ROC)^(54)^	Quasi- experimental	Schizophrenia group *n* 33	37·8, sd 1·7	*n* 14 female	Schizophrenia	Wt reduction programme: psychosocial evaluation and behaviour therapy. Healthy eating and calorie reduction. Exercise five times a week at mild–moderate intensity. Diet/exercise diaries Setting a hospital day care unit	N/A	BMI (kg/m^2^) Wt Waist Waist:hip ratio	Follow-up 10 weeks Participants *n* 33 Significant decreases from baseline to follow-up in BMI, wt and waist circumference (*P* < 0·01) and in waist to hip ratio (*P* < 0·05). Adherence: unreported.
Barnard-Kelly (2022) (UK) (From Whicher, 2021)	Nested qualitative (from RCT Whicher, 2021)	Baseline interview *n* 16 Follow-up interview *n* 10	21–64	N/A	Schizophrenia, schizoaffective disorder or first episode psychosis	Liraglutide as an adjuvant to promote wt loss. Setting: Mental health centres and primary care	Semi-structured interviews. Content and thematic analyses.	*Key themes* • Medication-associated weight gain • Impact of study on quality of life • Study information and support from trial was well received • Practical aspects of clinic attendance • Healthcare professional perspective	

RCT, randomised controlled trial; IG, intervention group; CG, comparator group; Wt, weight; QoL, quality of life.

Of the primary outcomes which were the focus of this systematic review, BMI was included in seventeen studies^(44–51,53,54,58,59)^ and body weight in fourteen studies^(44–47,49–51,53,54,56–59,61)^. Of the secondary outcomes, four studies included measures of QoL^(45,46,51,55)^, five of mental health outcomes^(47,50,51,55,61)^, ten of waist circumference^(44,47,50,51,54–57,59,61)^ and seven of other anthropometric measures^(44,50,51,54,57–59)^. Two studies included male-only participants^(44,45)^, two studies included female-only participants^(49,57)^ and one study did not report sex ratio^(48)^. Of the total number of participants, 52 % (*n* 684) were female. The majority of studies reported on patients diagnosed with schizophrenia (*n* 11)^(45,46,50–54,56–59)^ followed by bipolar disorder (*n* 2)^(47,48)^, borderline personality disorder (*n* 1)^(49)^, and schizophrenia and bipolar disorder (*n* 1)^(55)^, and three studies included participants with a range of SMI^(44,60,61)^.

Four study settings were on hospital grounds or inpatients^(46,54,57,59)^, eleven were community or outpatients^(47,48,50–53,55,56,58,60,61)^, and three studies did not report the settings^(44,45,49)^. The length of interventions ranged from 6 weeks^(51)^ to 5 years^(52)^. There were two qualitative studies^(53,60)^; one^(53)^ included a nested qualitative design but did not provide quotations; therefore, it was not possible to perform any qualitative analysis on this study.

### Intervention components

Interventions were diverse and included psychological interventions, two of which were compared in the same study (*n* 3)^(49,52)^ information giving (*n* 1)^(56)^, physical activity (*n* 2)^(44,45)^, and pharmacology (two interventions within one study) and one which was an experimental study with a nested qualitative element (*n* 5)^(47,50,51,60,61)^ or multi-component interventions (*n* 9)^(46,48,53–59)^ (Table 1).

### Psychology

Katekaru, Minn and Pobutsky^(52)^ trialled behavioural counselling during *ad hoc* check-up appointments. Participants were encouraged to attend local wellness classes that provided nutrition and exercise guidance. Galle *et al.* ^(49)^ implemented two interventions for bariatric surgery candidates, one focusing on interpersonal relationships and the other on behaviour change.

### Information giving

The first of two interventions in the study by Sugawara *et al.* ^(56)^ included brief advice on body weight with weigh-in sessions delivered by psychiatrists.

### Physical activity

Abdel-Baki *et al.* ^(44)^ assessed the feasibility of individual aerobic interval training, and Battaglia *et al.* ^(45)^ implemented soccer training sessions with an aim of improving the psychophysical condition of participants including QoL, body weight and physical performance.

### Pharmacology

Henderson *et al.* ^(50)^ investigated the effect of sibutramine for weight loss and in a further study^(51)^ compared the effect of ziprasidone as an adjunct treatment for olanzapine and for clozapine. Elmslie *et al.* ^(47)^ investigated carnitine supplementation, and Whicher *et al.* ^(61)^ compared liraglutide as an adjuvant to promote weight loss compared with placebo.

### Multi-component

Centorrino *et al.* ^(46)^ investigated a combination of behaviour, diet and exercise which included both aerobic and strength training. Kuo *et al.* ^(54)^ included a weight reduction intervention as part of a wider study and included behaviour therapy, exercise and dietary elements. Other multi-component interventions included structured behaviour change classes, healthy eating, physical activity and smoking cessation^(48)^; group education, exercise and social community activities^(53)^; physical activity in local streets, nutrition and tailored weight reduction plans^(57)^; education, physical activity and diet^(55)^; and tailored dietary plans and physical activity^(59)^. The second intervention in the study by Sugawara *et al.* ^(56)^ built on the first intervention by including a structured food and nutrition programme delivered by dietitians. Another study used behavioural strategies including barriers to change, problem-solving and goal setting with additional focus on diet and activity^(58)^.

### Comparator characteristics

The majority of CG were TAU, but three of these provided no detail^(56,58,59)^. In other studies, TAU involved medical check-ups, psychological evaluation and meetings with a bariatric surgeon^(49)^ and regular psychiatrist check-ups^(55)^. In the soccer intervention, the CG were instructed not to perform any organised physical activity during the experimental period^(45)^. In three of the pharmacological interventions, the CG was administered a placebo capsule^(47,50,61)^ and in the fourth there was no CG^(51)^. The CG in Frank *et al.* ^(48)^ was described as high-quality medical monitoring. In one study, the CG received the same treatment as the intervention group but did not have an SMI diagnosis^(57)^.

### Quality appraisal

A summary table of the CASP results is presented in Appendix 3.

### Randomisation and blinding

Of the ten RCT, four did not report the method of randomisation^(50,57–59)^ and six did not report the method of allocation concealment^(45,50,56–59)^. Three RCT reported comprehensive blinding of participants, study investigators and outcome assessors^(45,50,61)^.

## Results

In seven of the included studies, five studies performed an intention-to-treat analysis using Last Observation Carried Forward (LOCF)^(47,50,51,55,61)^. Of the studies that did not perform an intention-to-treat analysis, ten reported results in favour of the intervention^(45,46,48,49,52–54,56,58,59)^. Additionally, one of the studies had a high drop-out rate (29 %) and lost seven participants for reasons that pertained to the intervention^(56)^.

### Generalisability of the results

All studies addressed weight-related issues in adults with SMI and overweight or obesity as per our inclusion criteria. However, the intervention by Galle *et al.* ^(49)^ was tailored to bariatric patients, and Wu *et al.* ^(59)^ assessed obesity as BMI > 27 kg/m^2^ using Asian population standards, as such these two interventions may lack sufficient generalisability.

### Data synthesis

#### BMI (kg/m^2^)

Of the ten RCT, three studies reported BMI change score^(47,59,61)^, three reported BMI follow-up score^(45,50,55)^, and one reported both BMI follow-up and change score^(57)^. Two studies reported BMI outcomes as percentages and as such were not included in statistical synthesis^(48,58)^. Sugawara *et al.* ^(56)^ conducted a multi-arm study; as double counting of a CG is not recommended, the two interventions were combined (Doctor’s Weight Loss Advice group and Nutrition Education group) using a recommended formulae for combining summary statistics via Review Manager 5.3 calculator^(41)^. Additionally, Wu *et al.* ^(59)^ assessed obesity using a different standard to that of our inclusion criteria, as such this study was also excluded from pooled synthesis. In 2010, sibutramine, an appetite suppressant which had previously been used for the treatment of obesity, was suspended by the EU due to associated cardiovascular risks^(62)^. The pharmacological study by Henderson^(50)^ which trialled sibutramine was therefore excluded from analysis.

**Fig. 2.**
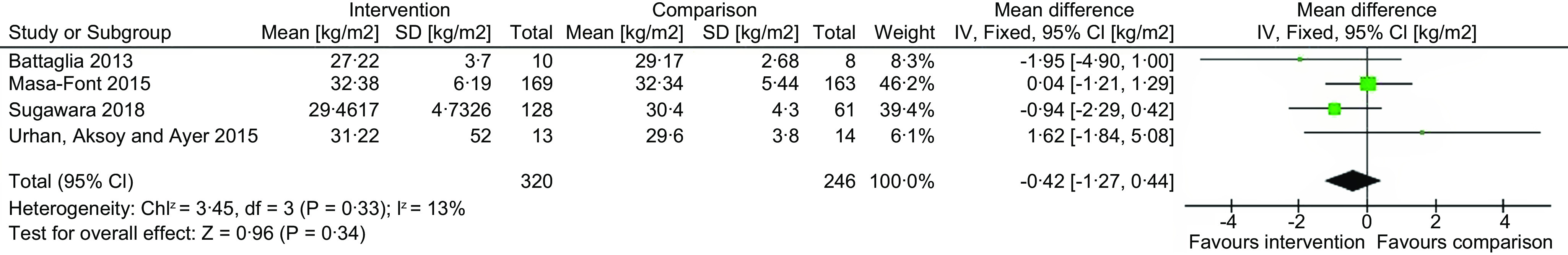
Pooled results of BMI outcomes from four RCT for intervention *v*. comparator groups.

The pooled data for mean differences in BMI at follow-up for four studies^(45,55–57)^ (*n* 566) were calculated via meta-analysis using a fixed effects model as there was evidence of low statistical heterogeneity (I^2^ = 13 %, *P* = 0·33). The results showed no overall effect in favour of intervention or comparison (MD –0·42, 95 % CI –1·27, 0·44, *P* = 0·34) (Fig. 2). As only four studies were included, pre-specified subgroup analysis was not performed.

Only four of the of the quasi-experimental studies reported mean BMI (sd)^(44,46,49,51)^, two of which compared two interventions^(49,51)^; therefore, pooled analysis of outcomes was deemed inappropriate. Mean differences (95 % CI) were instead calculated using pre-post results^(44,46,49,51)^. Only one study^(49)^ indicated a very large effect for both interventions at follow-up (interpersonal therapy group MD 14·20, 95 % CI, 12·19, 16·21; dialectical behavioural group MD 9·40, 95 % CI, 7·32, 11·48).

#### Body weight

**Fig. 3.**
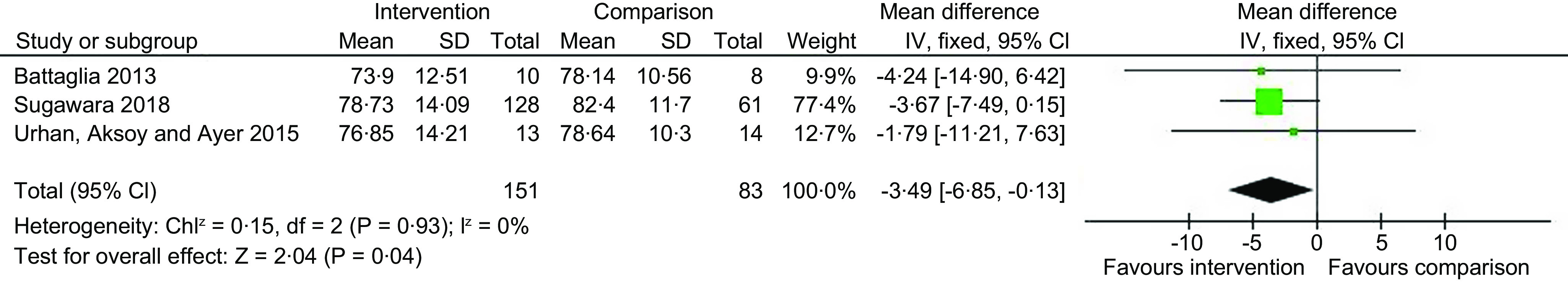
Pooled results of body weight outcomes from three RCT for intervention *v*. comparator groups.

The pooled data for mean differences in body weight (kg) at follow-up for three studies (*n* 234)^(45,56,57)^ were calculated via meta-analysis using a fixed effects model as there was evidence of low statistical heterogeneity (I^2^ = 0 %, *P* = 0·93). The results indicated a small effect in favour of the intervention (SMD –3·49, 95 % CI –6·85, –0·13, *P* = 0·04), although the upper boundary of the CI of all three studies indicated some uncertainty in the effect size (Fig. 3).

Of the quasi-experimental studies, only three reported mean (sd) and one study reported two pharmacological interventions^(44,46,51)^; individual measures of effect were calculated but showed no pre-post effect for the interventions in terms of reductions in weight.

Due to insufficient data to conduct meta-analyses, secondary outcomes were assessed using the results reported by individual study authors.

#### Quality of life

For QoL, one study^(45)^ found in favour of the intervention for the SF-12 Mental Component Score and the SF-12 Physical Component Score at 12-week follow-up (*P* < 0·0001), while a further study author^(55)^ reported in favour of the intervention for the SF-36 Standardized Physical Component Scale (Intervention 1·83, 95 % CI 0·70, 2·95 and CG 0·24, 95 % CI –0·74, 1·22) but in favour of the CG for the Standardized Physical Component Scale (Intervention –0·39, 95 %CI: –1·97, 1·19 and CG 2·19, 95 % CI 0·58, 3·81). While one study^(46)^ reported no significant differences in the Quality-of-Life Questionnaire from baseline to follow-up but omitted the results of the SF-36.

#### Impact on mental health

For mental health outcomes, three studies^(50,55)^ reported no effect for either the intervention or CG. Similarly, one quasi-experimental study^(51)^ also found no significant differences for any mental health outcome (The Positive and Negative Syndrome Scale, The Hamilton Depression Rating Scale and The Scale for the Assessment of Negative Symptoms) for either intervention group (clozapine or olanzapine groups).

#### Body composition

There were contrasting results across measures of body composition. One study^(57)^ reported that both the intervention and CG showed decreases in waist-to-hip ratio, while in contrast another study^(50)^ found a significantly greater increase in waist-to-hip ratio in the intervention group (*P* = 0·07). A third study^(58)^ found no significant between groups differences in waist-to-hip ratio. While one quasi-experimental study^(54)^ found a significant decrease in waist-to-hip ratio at follow-up (*P* < 0·05).

Two studies^(56,57)^ reported significantly greater decreases in waist circumference in favour of the CG (*P* < 0·05), while in contrast a further study^(50)^ reported significantly greater decreases in waist circumference in favour of the intervention (*P* < 0·005). A third study^(55)^, found slight mean increases in the intervention group (0·98, 95 % CI, 0·01, 1·95).

There were also differences across the quasi-experimental studies where two studies^(44,54)^ reported a significant decrease in waist circumference at follow-up (*P* < 0·01). In contrast, a further study^(51)^ found no significant decreases in waist circumference at follow-up in either the clozapine or olanzapine groups but a clear increase within the olanzapine group.

#### Qualitative result

As only one qualitative study was included^(61)^, overall thematic analysis was not possible. Within this individual study, semi-structured interviews explored expectations and experiences of taking part in the RCT, in addition to broader experiences of attempted weight loss. Within the study, thematic analysis reported by the authors showed that participants had pre-trial reservations about the liraglutide injections but reported no post-trial issues with this^(60)^. Other reported themes related to: medication associated weight gain; an improvement in the QoL as a result of the study; study information and support from trial being well received by the participants and practical aspects of attending the clinic such as issues with travel^(60)^.

## Discussion

### Summary of findings

The aim of this mixed methods systematic review was to assess the available evidence for the effectiveness of weight management interventions for people with SMI and overweight or obesity, effective elements of weight management interventions and to collate qualitative evidence of acceptability. Eighteen studies, representing nineteen diverse interventions were included in the systematic review, and four RCT were included in two meta-analyses^(45,55–57)^. As the number of studies included in the meta-analyses was small, subgroup analysis could not be conducted, and therefore the research question regarding which elements of interventions are effective was not answered. It was also not possible to conduct a meta-analysis of included quasi-experimental studies due to methodological heterogeneity.

While one meta-analysis of three studies in this systematic review showed a small effect in favour of the interventions in terms of reduction in body weight, the other meta-analysis found no effect for BMI. Individual study authors reported mixed results for anthropometric and QoL outcomes and no improvement in mental health outcomes. Only one qualitative study^(60)^ was included in the systematic review which was nested within an RCT^(61)^; therefore, the question of acceptability could not be addressed.

### Interpretation of the results in the context of other evidence

This systematic review and meta-analysis indicated that weight management interventions for people with overweight or obesity and SMI had a small effect on decreases in body weight. However, this finding was based on only three studies^(45,56,57)^. Furthermore, combined summary statistics were calculated for two interventions within one of the studies which was a multi-arm RCT^(56)^, and this resulted in an imbalance between the number of participants in the intervention arm (*n* 128) *v*. the control arm (*n* 61). As such this finding should be treated with caution. However, this finding is also consistent with other systematic reviews on weight management interventions for people with SMI. For example, one systematic review and meta-analysis focusing on pharmacological interventions for antipsychotic weight gain also found in favour of the intervention for decreases in body weight (MD –3·12, 95 % CI, –4·03, –2·21); although in contrast to this systematic review a small effect for BMI outcomes was also found (MD –0·94, 95 % CI, –1·45, –0·43)^(28)^, and this was based on sixteen studies as opposed to only three that were included in this meta-analysis. In another systematic review of weight management interventions for people with schizophrenia, cognitive behavioural therapy interventions were found to have a modest effect on weight reduction (WMD –1·69 kg CI –2·8, –0·6), and this was also based on only three studies^(37)^. It was highlighted that inconsistency in the reporting of results by individual study authors impacted the scope for analysis^(37)^. Inadequate reporting of results by study authors was also a barrier to more comprehensive analysis within this systematic review, for example, where outcomes were reported as percentages^(48,58)^ rather than mean (sd).

The three studies^(45,56,57)^ included in the meta-analysis of body weight outcomes for this systematic review included physical activity components. This observation may be based on only three studies but is potentially interesting particularly as evidence suggests that people with SMI partake in lower levels of physical activity than the general population which is due to a range of factors such as low mobility^(14,63)^ and physical health problems^(64)^. Of the nineteen interventions included in this systematic review, twelve included physical activity components^(44–48,52–55,57–59)^, but none described taking mobility or physical health challenges into consideration as a potential barrier to physical activity. For example, the soccer practice intervention^(45)^ involved moderate to vigorous activity^(45)^, and a further intervention included walking up and downstairs^(59)^ potentially excluding people with SMI and mobility challenges. This perhaps highlights the importance of tailoring weight management interventions to SMI.

This systematic review identified only one eligible qualitative study and as such could not address intervention acceptability; a previous systematic review of interventions for people with bipolar disorder and obesity also reported a lack of qualitative evidence in this area^(65)^. Qualitative evidence can add meaning to findings^(66)^. For example, Davidson^(67)^ questioned patients with SMI about their experiences of an intervention to reduce readmission to hospital and found that the study had mistakenly focused on addressing the dysfunctions associated with SMI rather than the social difficulties following discharge that made hospitalisation seem the preferred option for some patients with SMI^(67)^. Other qualitative research involving people with SMI has highlighted the wider socio-economic challenges of managing weight^(68)^ including feelings of stigmatisation and isolation^(14,66)^, food insecurity and a lack of long-term support^(13)^, while for those in an inpatient setting loss of control and a sense of confinement^(66)^. The one qualitative study included in this systematic review highlighted the acceptability of text message reminders for appointments to receive liraglutide injections and concerns about the potential side effects of the medication^(61)^. Text message reminders have been shown to increase aherence to treatment^(69)^ and reduce missed community mental health appointments by up to 28 %, which is important as this has a potential national cost-saving benefit of an estimated £150 million a year^(70)^.

### Limitations of the evidence included in the review

The NICE recommends using waist circumference in addition to BMI for people with a BMI < 35 kg/m^2(71)^, while the WHO recommends the use of waist circumference alone or in conjunction with BMI^(72)^; waist circumference is an indicator of body fat accumulation around the abdominal area which is associated with obesity^(37,65)^. Combined waist circumference measurements and BMI also provide estimated cut-off points for disease risk associated with overweight and obesity^(72)^. However, only ten studies included in this systematic review reported both BMI and waist circumference. The secondary outcomes in this review did include other anthropometric measures of adiposity, but with mixed results as reported by study authors.

The Standard Evaluation Framework for weight management interventions^(65)^ suggests that data relating to the success of weight management interventions is patchy and inconsistent, as has been seen within this systematic review; one reason being that inappropriate measures are often used^(65)^. Complementary weight management-related outcomes could be included in studies, such as the proportion of participants who achieve 5 to 10 % reductions in body weight. Research has shown that weight loss of between 5 and 10 % reduces cardiovascular risk factors for the general population^(66)^ and has also been recommended by Public Health England as an outcome measure^(65)^. Within this systematic review, one study included this as an outcome^(61)^ reporting that at 3-month follow-up, *n* 8 (50 %) of the intervention group had experienced weight reductions of more than 5 % compared with only *n* 1 (5 %) of the control group; perhaps indicating this as a potential measure of short-term weight loss for studies of limited duration. Other additional outcomes potentially include assessing changes in intake of fruit and vegetables^(65)^ as SMI is associated with lower dietary quality such as inadequate fruit and vegetable consumption and higher intakes of takeaways than the general population^(67)^.

A lower cut-off point than the standard threshold value (BMI 25 kg/m^2^) is recommended by NICE Guidelines for Black and Asian groups as these populations are at an increased risk of chronic health conditions at a lower BMI compared with White populations^(68)^. Only one study included in this systematic review adjusted BMI thresholds using an Asian standard at a lower threshold^(59)^. Only four studies reported ethnicity^(50,51,58,60)^; Henderson reported more than a quarter (27 %) of participants were of African American ethnicity but did not adjust BMI thresholds. Only one study included in the meta-analysis for BMI outcomes reported ethnicity, but this was reported as ‘other ethnic group’ (13 %) which lacked clarity^(61)^. Evidence shows that ethnic background can impact weight loss outcomes^(73,74)^, as such had there been adequate data, subgroup analysis by ethnic background may have been of benefit to determine whether ethnicity impacts weight-related outcomes for people with SMI. This is particularly important as evidence shows that people from Black and ethnic minority groups are overrepresented in psychiatric inpatient settings compared with other people with SMI^(14)^ and may have worse mental health outcomes^(63,75)^.

### Limitations of the review processes used

Although a thorough search of databases was conducted, due to time constraints sources of grey literature did not form part of the search strategy for this systematic review. Grey literature refers to articles not published by a commercial reviewer such as those produced by governments and organisations^(76,77)^. Inclusion of such material in a systematic review is considered good practice for minimising the risk of publication bias where publication of research findings is influenced by the direction of results^(78)^ as evidence shows that studies reporting positive outcomes are more likely to be published which can cause overestimation of effect sizes in meta-analyses^(76)^. However, there is also some criticism surrounding the search engines commonly used for grey literature searches such as Google Scholar and Web of Science. One study on the role of Google Scholar in the search for grey literature highlighted constraints in search string complexity^(76)^ which could potentially limit search results. Additionally, some systematic reviewers restrict grey literature searches to the first one hundred records, an activity that has been described as not evidence based and disproportionate to the volume of records found via other databases^(76)^. This is of particular concern as Google Page Rank, lists results according to popularity^(77)^. In addition to these drawbacks, despite guidance on where to find grey literature^(77)^ there is also a lack of standardised methodology^(77)^.

Best practice is for reviewers to independently extract data; however, this was not possible as the systematic review formed part of a wider 12-month project. However, to ensure reliability and accuracy of the screening process, four reviewers independently checked extracted data.

### Implications for practice and policy

This systematic review was inconclusive as inadequate evidence was found to inform practice or policy on weight management for people with SMI and overweight or obesity as there is an absence of a rigorous evidence particularly in terms of the acceptability of interventions.

### Future research

The results of this review have highlighted a severe lack of qualitative studies specifically looking at the experiences of adults with SMI participating in weight management interventions. Therefore, future experimental studies should focus on mixed methods approaches that incorporate a qualitative element such as interviews and focus groups to capture further insight into the barriers and facilitators to successful weight management. Participant feedback on weight management interventions has been advocated by Public Health England as an essential opportunity to identify the strengths and weaknesses of interventions^(65)^.

It is recommended that studies include other measures of weight loss such as measures of central adiposity or 5 to 10 % weight loss as outcome measures in addition to weight and BMI. BMI can be limited as it does not account for fat and fat-free mass^(79)^ and has been shown to misclassify participants. A comparison between bioelectric impedance analysis (BIA) and BMI as outcome measures in men with obesity and schizophrenia showed that BMI calculations misclassified men who had 30 % body fat as having a healthy weight rather than having obesity^(80)^.

Finally, there was inadequate reporting within some of the studies included in this review. Consistency and completeness in the reporting of primary studies would give more scope for performing a meta-analysis. To ensure optimal reporting primary study, authors should follow standardised guidelines for the reporting of primary studies such as Consolidated Standards of Reporting Trials (CONSORT)^(81)^. This was also noted by another systematic reviewer of weight reduction interventions for people with schizophrenia^(37)^.

### Conclusions

People living with SMI have higher rates of overweight and obesity than the general population. This mixed methods systematic review aimed to assess both quantitative and qualitative evidence on weight management interventions for adults living with SMI and overweight or obesity. There was a lack of qualitative evidence and a small effect for body weight reduction based on three studies but no effect on BMI. It is recommended that future primary studies integrate qualitative methodology into experimental study design to capture participants’ experiences of weight management and follow standardised guidelines to enable complete and transparent reporting. It is also recommended that additional outcome measures be used to complement weight and BMI outcomes such as measures of central adiposity and reductions in 5 to 10 % of body weight or changes in dietary quality.

## Appendix 1 PRISMA twenty-seven-item checklist for transparent reporting of systematic reviews of healthcare interventions.

**Table:**

Section and topic	Item #	Checklist item	Location where item is reported
Title			pp.
Title	1	Identify the report as a systematic review.	1
Abstract			
Abstract	2	See the PRISMA 2020 for Abstracts checklist.	1
Introduction			
Rationale	3	Describe the rationale for the review in the context of existing knowledge.	2–3
Objectives	4	Provide an explicit statement of the objective(s) or question(s) the review addresses.	3
Methods			
Eligibility criteria	5	Specify the inclusion and exclusion criteria for the review and how studies were grouped for the syntheses.	4
Information sources	6	Specify all databases, registers, websites, organisations, reference lists and other sources searched or consulted to identify studies. Specify the date when each source was last searched or consulted.	3
Search strategy	7	Present the full search strategies for all databases, registers and websites, including any filters and limits used.	Appendix 2
Selection process	8	Specify the methods used to decide whether a study met the inclusion criteria of the review, including how many reviewers screened each record and each report retrieved, whether they worked independently, and if applicable, details of automation tools used in the process.	4
Data collection process	9	Specify the methods used to collect data from reports, including how many reviewers collected data from each report, whether they worked independently, any processes for obtaining or confirming data from study investigators, and if applicable, details of automation tools used in the process.	4
Data items	10a	List and define all outcomes for which data were sought. Specify whether all results that were compatible with each outcome domain in each study were sought (e.g. for all measures, time points and analyses), and if not, the methods used to decide which results to collect.	4–5
	10b	List and define all other variables for which data were sought (e.g. participant and intervention characteristics, and funding sources). Describe any assumptions made about any missing or unclear information.	4
Study risk of bias assessment	11	Specify the methods used to assess risk of bias in the included studies, including details of the tool(s) used, how many reviewers assessed each study and whether they worked independently, and if applicable, details of automation tools used in the process.	4
Effect measures	12	Specify for each outcome the effect measure(s) (e.g. risk ratio and mean difference) used in the synthesis or presentation of results.	4
Synthesis methods	13a	Describe the processes used to decide which studies were eligible for each synthesis (e.g. tabulating the study intervention characteristics and comparing against the planned groups for each synthesis (item #5)).	4
	13b	Describe any methods required to prepare the data for presentation or synthesis, such as handling of missing summary statistics, or data conversions.	n/a
	13c	Describe any methods used to tabulate or visually display results of individual studies and syntheses.	4
	13d	Describe any methods used to synthesise results and provide a rationale for the choice(s). If meta-analysis was performed, describe the model(s), method(s) to identify the presence and extent of statistical heterogeneity, and software package(s) used.	4
	13e	Describe any methods used to explore possible causes of heterogeneity among study results (e.g. subgroup analysis and meta-regression).	4
	13f	Describe any sensitivity analyses conducted to assess robustness of the synthesised results.	4
Reporting bias assessment	14	Describe any methods used to assess risk of bias due to missing results in a synthesis (arising from reporting biases).	n/a
Certainty assessment	15	Describe any methods used to assess certainty (or confidence) in the body of evidence for an outcome.	n/a
Results			
Study selection	16a	Describe the results of the search and selection process, from the number of records identified in the search to the number of studies included in the review, ideally using a flow diagram.	5–6
	16b	Cite studies that might appear to meet the inclusion criteria, but which were excluded, and explain why they were excluded.	n/a
Study characteristic	17	Cite each included study and present its characteristics.	7–18
Risk of bias in studies	18	Present assessments of risk of bias for each included study.	18; Appendix 3
Results of individual studies	19	For all outcomes, present, for each study: (a) summary statistics for each group (where appropriate) and (b) an effect estimate and its precision (e.g. confidence/credible interval), ideally using structured tables or plots.	18–19
Results of syntheses	20a	For each synthesis, briefly summarise the characteristics and risk of bias among contributing studies.	n/a
	20b	Present results of all statistical syntheses conducted. If meta-analysis was done, present for each the summary estimate and its precision (e.g. confidence/credible interval) and measures of statistical heterogeneity. If comparing groups, describe the direction of the effect.	18–19
	20c	Present results of all investigations of possible causes of heterogeneity among study results.	n/a
	20d	Present results of all sensitivity analyses conducted to assess the robustness of the synthesised results.	n/a
Reporting biases	21	Present assessments of risk of bias due to missing results (arising from reporting biases) for each synthesis assessed.	n/a
Certainty of evidence	22	Present assessments of certainty (or confidence) in the body of evidence for each outcome assessed.	n/a
Discussion			
Discussion	23a	Provide a general interpretation of the results in the context of other evidence.	21–22
	23b	Discuss any limitations of the evidence included in the review.	22–23
	23c	Discuss any limitations of the review processes used.	23
	23d	Discuss implications of the results for practice, policy and future research.	23
Other information			
Registration and protocol	24a	Provide registration information for the review, including register name and registration number, or state that the review was not registered.	3
	24b	Indicate where the review protocol can be accessed, or state that a protocol was not prepared.	3
	24c	Describe and explain any amendments to information provided at registration or in the protocol.	n/a
Support	25	Describe sources of financial or non-financial support for the review, and the role of the funders or sponsors in the review.	n/a
Competing interests	26	Declare any competing interests of review authors.	24
Availability of data, code and other materials	27	Report which of the following are publicly available and where they can be found: template data collection forms; data extracted from included studies; data used for all analyses; analytic code; any other materials used in the review.	5

## Appendix 2 Search strategy.

**Table:**

Population:
**S1:** ‘severe mental illness’ OR ‘serious mental illness’ OR ‘enduring mental illness’ OR schizophrenia OR Schizophrenic OR psychosis OR ‘psychotic disorder’ OR ‘severe depression’ OR ‘major depression’ OR ‘manic depression’ OR ‘manic depressive disorder OR bipolar OR ‘delusional disorder’ OR ‘personality disorder’ **S2:** obes* OR overweight OR ‘over weight’ OR weight OR BMI OR ‘body mass index’ OR ‘waist circumference’ **S3:** adult* **S4: S1 AND S2 AND S3** **Interventions:** **S5:** ‘physical activity’ OR exercise OR fitness OR ‘ e health’ or ‘m health’ OR sedentary **S6:** Pharmacol* OR Metformin OR Orlistat OR Liraglutide **S7:** Nutrition OR ‘specialist weight’ OR diet OR food **S8:** Mindful* AND (eating OR diet) **S9:** Health* AND (‘eating culture’ OR lifestyle OR ‘life style’ OR improvement OR living) **S10:** Psychol* OR ‘cognitive behavio*’ **S11:** Intervention OR Education OR information OR support OR leaflets OR program* OR management **S12:** ‘Behavio* change’ OR attitudes OR perception* Carry out the following: **S13:** Pop + 5 + 11 **S14:** Pop + 5 + 11 + 12 **S15:** Pop + 6 + 11 **S16:** Pop + 6 + 11 + 12 **S17:** Pop + 7 + 11 **S18:** Pop + 7 + 11 + 12 **S19:** Pop + 8 + 11 **S20:** Pop + 8 + 11 + 12 **S21:** Pop + 9 + 11 **S22:** Pop + 9 + 11 + 12 **S23:** Pop + 10 + 11 **S24:** Pop + 10 + 11 + 12 S13 or S15 or S17 or S19 or S21 or S23 S14 or S16 or S18 or S20 or S22 or S24

## Appendix 3 Summary of CASP results for included studies. RCT, randomised controlled trial.

**Table:**

CASP Critical Appraisal Tool														
Author, Year	Study type	Q1	Q2	Q3	Q4a	Q4b	Q4c	Q5	Q6	Q7	Q8	Q9	Q10	Q11
Abdel Baki, 2013	Quasi-experimental	Y	N	N	N	N	Can’t Tell	Y	N/A	N	Y	Can’t Tell	Can’t Tell	Can’t Tell
Barnard-Kelly, 2022	Qualitative	Y	Y	Y	Y	N/A	N/A	Y	Can’t tell	Y	Y	Y	N/A	N/A
Battaglia, 2013	RCT	Y	Y	Y	T	Y	Y	Y	Y	Y	Y	Can’t Tell	Can’t Tell	Can’t Tell
Centorrino, 2006	Quasi-experimental	Y	N	Y	N	N	Can’t Tell	N/A	N/A	N	Y	Can’t Tell	Can’t Tell	Can’t Tell
Elmslie, 2006	RCT	Y	Y	Y	Y	Y	Can’t Tell	Y	Y	Y	Y	Can’t Tell	Can’t Tell	Can’t Tell
Frank, 2015	RCT	Y	Y	N	Can’t Tell	Y	Y	Y	Y	Y	Y	Can’t Tell	Can’t Tell	Can’t Tell
Galle. 2017	Quasi-experimental	Y	N	Y	N	N	Can’t Tell	Y	Y	Y	Y	Can’t Tell	N	Can’t Tell
Henderson, 2005	RCT	Y	Y	Y	Y	Y	Can’t Tell	Y	Y	Y	Y	Y	Can’t Tell	Can’t Tell
Henderson, 2009	Quasi-experimental	Y	N	N	N	N	N	Y	Y	Y	Y	Can’t Tell	Can’t tell	Can’t Tell
Katekaru, 2015	Quasi-experimental	Y	N	Y	N	N	Y	Can’t Tell	N/A	N	N	Can’t Tell	Can’t tell	Can’t Tell
Klam, 2016	Quasi-experimental	Y	N	Y	N	N	Can’t Tell	N/A	N/A	N	N	Can’t Tell	Can’t Tell	Can’t Tell
Kuo, 2013	Quasi-experimental	Y	N	Y	Can’t Tell	Can’t Tell	Can’t Tell	Y	Y	Y	Y	Can’t Tell	Can’t Tell	Can’t Tell
Masa-Font, 2015	RCT	Y	Y	Y	Can’t Tell	Y	Y	Y	Y	Y	Y	Can’t Tell	Can’t tell	Can’t Tell
Sugawara, 2018	RCT	Y	Y	Y	N	N	Can’t Tell	Y	Y	N	Y	Can’t Tell	Can’t Tell	Can’t Tell
Urhan, 2015	RCT	Y	N	Y	Can’t Tell	Can’t Tell	Can’t Tell	Y	Can’t Tell	Y	Y	Can’t Tell	Can’t Tell	Can’t Tell
Weber, 2006	RCT	Y	Y	Y	Can’t Tell	Can’t Tell	Y	Can’t Tell	Y	N	Y + N	Can’t Tell	Can’	Can’t Tell
Whicher, 2021	RCT	Y	Y	N	Y	Y	Y	Y	Y	Y	Y	Y	Y	Can’t tell
Wu *et al.*, 2007	RCT	Y	Y	Y	Can’t Tell	Can’t Tell	Can’t Tell	Can’t Tell	Can’t Tell	Y	Y	Can’t Tell	Can’t Tell	Can’t Tell
